# Novel SLC34A3 mutation causing hereditary hypophosphataemic rickets with hypercalciuria in a Gambian family^☆^

**DOI:** 10.1016/j.bone.2012.12.003

**Published:** 2013-03

**Authors:** Vickie Braithwaite, John M. Pettifor, Ann Prentice

**Affiliations:** a MRC Human Nutrition Research, Elsie Widdowson Laboratory, Cambridge, UK; b MRC/Wits Developmental Pathways for Health Research Unit, Faculty of Health Sciences, University of the Witwatersrand, Johannesburg, South Africa; c MRC Keneba, Keneba, West Kiang, The Gambia

**Keywords:** HHRH, Rickets, SLC34A3, Gene mutation, Africa

## Abstract

Three siblings, aged 12, 4 and 2 years, presented at a Gambian clinic with bone deformities. Radiographs of knees and wrists confirmed the presence of florid rickets. The family (including 2 unaffected siblings and the mother) were investigated for hereditary rickets.

The three affected siblings had biochemical features of hereditary hypophosphataemic rickets with hypercalciuria (HHRH) with normal plasma calcium and 25-hydroxyvitamin D concentrations, elevated 1,25-dihydroxyvitamin D, hypophosphataemia, hyperphosphaturia and hypercalciuria. At presentation, two of the three affected siblings had an elevated fibroblast growth factor-23 (FGF23) concentration. The mother and clinically unaffected siblings had largely normal biochemistry.

Genetic analysis of the SLC34A3 gene, encoding the type IIc sodium-phosphate cotransporter, in DNA samples from the siblings and their mother was conducted. Three single nucleotide polymorphisms (SNPs) S168F, E513V and L599L were identified. E513V and L599L had been previously identified as benign polymorphisms. S168F however, is a previously unreported variant. In silico mutation evaluation predicted that the S168F mutation causes changes in the protein product which are damaging to its function. In addition, the three clinically affected siblings were homozygous in the S168F variant whereas the unaffected family members were carriers.

This study describes a biochemical profile and complementary gene data consistent with a rare genetic hypophosphataemic rickets disease in a family from rural Gambia. To our knowledge, this study reports the first cases of HHRH in Africa and describes a novel causal mutation within the SLC34A3 gene.

## Highlights

► A Gambian family of 5 children where 3 siblings had florid rickets with suspected HHRH ► Biochemical profile of hypophosphataemia with elevated urinary phosphate and calcium excretion. ► Genetic analysis of the SLC34A3 gene indicated a novel mutation of S168F. ► The siblings with rickets were homozygous in S168F, the unaffected siblings were carriers. ► We report the first cases of hereditary hypophosphataemic rickets with hypercalciuria in Africa.

## Introduction

Children with putative dietary calcium deficiency rickets and chronically elevated circulating fibroblast growth factor-23 (FGF23), have been reported in The Gambia [1]. It has been proposed that chronically low dietary calcium (Ca) supply resulting in a 1,25-dihydroxyvitamin D (1,25(OH)_2_D)-driven increase in FGF23 concentration and consequent excessive urinary (*u*) phosphate (P) loss may be contributing to the aetiology of this form of rickets [1,2].

During a study to assess the prevalence of rickets in The Gambia, a family with apparent hereditary rickets was investigated [2]. Two siblings (S5* and S2*) with the same mother and father presented at a clinic in The Gambia with visible bone deformities and reported bone pain. Radiographs confirmed the presence of florid rickets. On further investigation, an additional younger sibling (S1*) with bone deformities was reported. Two other siblings (S3 and S4) were clinically normal as was the mother. The family was investigated for possible hereditary rickets, which revealed biochemical features of hereditary hypophosphataemic rickets with hypercalciuria (HHRH) in the three affected siblings (S5*, S2* and S1*). Mutations within the SLC34AC gene are known to cause HHRH [3–5]. Subsequent genotyping of the SLC34AC gene revealed a novel mutation which was homozygous in the three affected siblings. The mother and the other siblings were carriers for the same mutation.

This case series describes the biochemical profile of the siblings with rickets and subsequent candidate gene analysis of the family members (affected and unaffected) to establish aetiology. To our knowledge, this study reports the first cases of HHRH in Africa and describes a novel causal mutation within the SLC34A3 gene.

## Materials and methods

### Subjects

Three siblings (S5* female, S2* male and S1* male) had bone deformities (*) and were seen at a Gambian clinic on one or more occasions between 2000 and 2006. Their other siblings (S3 female and S4 female) and the parents of the siblings showed no signs of bone deformities. A family history revealed that, at the time, no-one else in the extended family had bone deformities and that the parents were not close relatives. However, it is possible that they are distantly related as consanguinity is not uncommon in this population. Age-matched data obtained from a community study, described in detail elsewhere [2], provided contemporaneous local reference data for anthropometry and biochemistry across appropriate age bands: 2.0–5.9 y (*n* = *10*), 6.0–9.9 y (*n* = *10*), 10.0–13.9 y (*n* = *10*), 14.0–17.9 y (*n* = *10*), and 18.0–47.0 *y* (*n* = *52*) (Table 1). Standard deviation scores (Z-scores) were calculated for each variable for each subject using the age-appropriate reference data in the following way:

$$\frac{{\left[\text{value}\right]}_{\mathrm{subject}}-{\left[\mathrm{mean}\right]}_{\mathrm{references}}}{{\mathrm{Standard deviation}}_{\mathrm{references}}}\text{.}$$

### Study approvals

Ethical approvals for the use of clinical notes and for the research study were obtained from The Gambian Government/MRC Laboratories Joint Ethics Committee. Written informed consent was obtained from the family. The father did not participate in the study.

#### Clinical assessment and anthropometry

A detailed clinical assessment was conducted to identify the presence of any clinical signs and symptoms of rickets including; enlarged wrists or ankles, leg pain, difficulty walking and bow-leg or windswept deformity, and to discount other diseases associated with bone deformities. Bilateral radiographs were taken of knees and wrists of the affected children and were scored by a consultant paediatrician (JMP) using a 10-point scoring system developed by Thacher et al. [6]. Standard anthropometry was conducted which included weight (wt) and standing height (ht).

#### Fasting blood and 2 h urine collection

An overnight-fasted, 2 h urine (*u*) sample was collected between the hours of 07.00 and 09.00. Acidified (HCl 10 μL/mL, laboratory reagent grade SD 1.18, Fisher Scientific UK Ltd., Loughborough, UK) urine aliquots were stored at − 20 °C and then later transported frozen on dry ice to MRC Human Nutrition Research (HNR), Cambridge, UK for analysis. A fasting, venous blood sample was collected, in the middle of the 2 h urine collection, transferred to lithium heparin (LiHep) and EDTA-coated tubes, plasma separated by centrifugation at 4 °C and frozen at − 20 °C, and later transported frozen on dry ice to MRC HNR, where the plasma samples and the blood cell pellets were stored at − 80 °C until analysis.

#### Biochemical analysis

The plasma samples were analysed for markers of vitamin D, Ca and P metabolism using commercially-available methods according to the manufacturers' instructions: intact PTH (Immunoradiometric assay; DiaSorin Ltd., Berks, UK), FGF23 (C-terminal ELISA; Immutopics Inc., CA, USA), 25OHD and 1,25(OH)_2_D (radioimmunoassay DiaSorin, MN, USA and IDS, Tyne and Wear, UK respectively). The following colorimetric methods (Cobras Fara, Roche Products Ltd, UK and Konelab™ Analyser 20i, Finland) were used to determine plasma analytes: total calcium (TCa) by methylthymol blue (Roche Unit-Kit II) and arsenazo III (Konelab™ 981367); P, ammonium molybdate (Roche Unit-Kit II and Konelab™ 981890); and total alkaline phosphatase (TALP), *p*-nitrophenyl phosphate at 37 °C (Roche Alp MPR2 and Konelab™ 981832). For FGF23, > 125 RU/mL was used as an upper-limit cut-off of normality. Acidified urine was used to determine urinary (*u*) *u*Ca and *u*P employing the same colorimetric methods as for plasma and *u*Cr was determined using the Jaffe method (Konelab™ 981832). *u*Ca excretion was expressed as a molar ratio with *u*Cr. Tubular maximal reabsorption of phosphate (TmP:GFR) (mmol/L) was determined in the following way: Tubular reabsorption of phosphate (TRP) = 1 − {(*u*P/P) × (Cr/*u*Cr)}, if TRP < 0.86 then TmP:GFR = TRP × P mmol/L, if TRP > 0.86 then TmP:GFR = (0.3 × TRP/{1 − (0.8 × TRP)}) × P mmol/L [7].

#### Genetic analysis

The frozen EDTA blood pellets were sent to the Laboratory for Molecular Diagnostics, Centre of Nephrology and Metabolic Disorders, Berlin, Germany for candidate gene analysis. DNA was extracted using standard methodology (QIAamp DNA kit, Qiagen, West Sussex, UK). Sequencing was performed using standard procedures with dye terminators. The fragments were separated by capillary electrophoresis (ABI Prism 3130 genetic analyser, Hitachi Ltd., CA, USA). The candidate gene (SLC34A3) was sequenced in 12 fragments consisting of one exon plus ~ 20 base pairs on either side of the exon in the youngest affected sibling (S1*). All single nucleotide polymorphisms (SNPs) were identified and the remaining siblings (*n* = 4) and mother were then screened for the variant SNPs. All SNPs were analysed using the NCBI (National Center for Biotechnology Information) database according to the GenBank transcript NM_080877.2. *In silico* mutation evaluation to predict protein structure, was conducted using two programmes: Mutation taster [8] and PolyPhen-2 [9]. Sequence alignment was performed using the Basic Local Alignment Search Tool (BLAST®) on the NCBI database.

## Results

### Presentations

Case 1 Sibling 5* (S5*)

S5* (female) was the eldest of the five siblings and first presented with knock-knee deformity and bone pain at the age of 12 y in July 2000. She was short and light for her age relative to local age matched children (Table 2). Biochemical analysis of a blood sample revealed that she had concentrations of 25OHD and Ca within the normal range, PTH was low with elevated concentrations of 1,25(OH)_2_D and TALP. In addition she had low plasma P with a normal concentration of FGF23. Urine analysis confirmed a low TmP/GFR and hypercalciuria. Radiographs confirmed S5* to have active rickets with a Thacher score of 4 and evidence of Looser zones and growth arrest lines.

Case 2 Sibling 2* (S2*)

S2* (male) presented with bow-leg deformity and bone pain at the age of 2 y in May 2001. He was short for his age. As with S5*, he had concentrations of 25OHD and Ca within the normal range, elevated concentrations of 1,25(OH)_2_D and TALP and low plasma P. Unlike S5*, he had an elevated FGF23. PTH was not measured due to insufficient plasma sample. TmP/GFR was reduced and *u*Ca:*u*Cr increased. Radiographs confirmed S2* to have active rickets with a Thacher score of 8.

Case 3 Sibling 1* (S1*)

S1* (male) was the youngest sibling (4 y) and first presented with bow-leg deformity in July 2006. Like S2*, he was short for his age, and reported bone pain. Similar to S5* and S2* he had concentrations of 25OHD and Ca within the normal range, elevated concentrations of TALP and low plasma P. Like S2*, he also had elevated FGF23. Neither PTH nor 1,25(OH)_2_D was measured. Urine analyses confirmed increased renal P and Ca excretion. Radiographs confirmed S1* to have active rickets with a Thacher score of 7.

### Characterisation of unaffected siblings (S3 and S4) and mother

S3 (F), S4 (F) and the mother of the children were seen in July 2006 and were aged 11, 15 and 35 y respectively (the father did not consent to examination or biochemical). They showed no clinical bone deformities, but no radiographs were taken to confirm this. S3 and S4 were both short and heavy for their ages. Their biochemical profiles were largely normal, however, S4 had a lower than average plasma P and TmP:GFR for her age, albeit not as low as her affected siblings and both the mother and S3 had a higher than average *u*Ca excretion (*u*Ca:*u*Cr). No signs or symptoms of nephrocalcinosis were reported in any of the siblings or the mother.

### Treatment of S5* and S2*

Prior to the completion of the investigations, S5* and S2* were treated with calcium and vitamin D with little or no clinical and radiological responses although biochemically 25OHD and 1,25(OH)_2_D concentrations did rise. Reviewing the results as a whole and especially the findings of a low TmP:GFR and high *u*Ca:*u*Cr, a tentative diagnosis of hereditary hypophosphataemic rickets with hypercalciuria (HHRH) was made and the two siblings were started on oral phosphate therapy. Plasma P increased somewhat in S2* but little change was seen in S5*. Problems with treatment compliance were acknowledged and the therapy was subsequently terminated.

#### Gene sequencing and screening

**Fig. 1:**
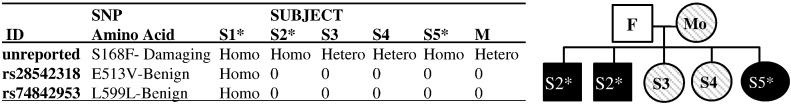
Single-nucleotide polymorphisms (SNPs) on the SLC34A3 (NM_080877.2) gene in siblings (S1-5) and their mother (M). Subjects with rickets are indicated by * and are shaded in black. Females are indicated by a circle and males by a square. Genetic analysis on the father (F) was not conducted. Homo = homozygous in the SNP, heterozygous = carriers of the SNP, 0 = no copy of the SNP.

Candidate gene analysis of the SLC34AC gene, encoding a type IIc sodium-phosphate transporter (NaPi-IIc) expressed in the kidney, was conducted in the DNA sample from the youngest affected sibling (S1*) (Fig. 1). Three SNPs were found for which the other family members were then screened. Two of the SNPs had been previously reported on the NCBI database and had been assigned the following reference numbers rs28542318 and rs74842953. These SNPs referred to a non-synonymous mutation E513V (c.1538A > T) and a synonymous mutation L599L (c.1795 T > C) respectively. *In silico* mutation analysis suggested that these two SNPs were benign polymorphisms and they were unique to S1* and were not present in the other investigated family members. The third SNP was a novel non-synonymous mutation resulting in an amino acid change S168F (c.503C > T). Blast alignment results indicated that the flanking amino acid regions at the site of the S168F mutation were highly conserved throughout species (Table 3). In silico mutation analysis predicted that this SNP would cause a damaging mutation in the NaPi-IIc protein, the likely cause being the loss of function of the transmembrane domain spanning 133–188 amino acids. Prediction analysis, suggested that the protein product containing S168F was of normal length (599 amino acids) and that the correct reading frame was maintained. The S168F mutation was present homozygously in the affected siblings (S5*, S2* and S1*) and heterozygously in the unaffected family members (Fig. 1).

## Discussion

The Republic of The Gambia (latitude 13°N) in West Africa has a hot and dry tropical climate with a single wet season from June to October. There is abundant UVB-containing sunshine throughout the year and a lifestyle that does not limit skin UVB-exposure. Cases of rickets have, however, been reported and have been attributed predominantly to a chronically low dietary calcium (Ca) intake leading to a 1,25(OH)_2_D-driven increase in FGF23 leading to urinary phosphate (P) wasting and rickets [1,2].

The family described in this study is, however, strikingly different to our previous reports of rickets in The Gambia. To our knowledge, this study documents the first cases of hereditary hypophosphataemic rickets with hypercalciuria (HHRH) in Africa. The cause of HHRH is a mutation within the gene encoding the Type IIc sodium-phosphate co-transporter [3,4]. There are two major NaPi co-transporters involved in P reabsorption in the proximal tubule of the kidney. These are NaPi-Type IIa and -Type IIc, both of which are regulated by FGF23 and PTH. Although NaPi-IIa is thought to account for up to 70% of renal P reabsorption [5], we show in this study that NaPi-IIc must also play an integral role in P homeostasis. Additionally, we describe a novel mutation in the SLC34AC gene.

HHRH is associated with a distinct biochemical profile resulting from the loss of function of NaPi-IIc. This includes a reduction in P reabsorption in the renal tubules leading to excessive urinary P loss. A low TmP:GFR and plasma P are characteristic of this syndrome which is often associated with an elevated 1,25(OH)_2_D and consequent hypercalciuria. A raised FGF23 is not a distinguishing feature of this syndrome, however we found elevated FGF23 at first presentation with rickets in 2 out of 3 cases. This may be explained by a chronically low dietary Ca intake and increased 1,25(OH)_2_D-driven increase in FGF23 as described in the majority of Gambian nutritional rickets [1]. Alternatively, this may have been due, in part, to the young age of these children (< 4 y) as we have previously seen that FGF23 tends to decrease throughout childhood (unpublished data). However the FGF23 Z-scores, calculated using local age-matched control children, were high at 2.5 and 1.2. It may also, however, be an indicator of poor iron status leading to an increased expression of the FGF23 gene and a subsequent increase in degradation of the intact FGF23 hormone [10]. However, this possibility cannot be explored in greater detail as the C-terminal assay and not the Intact FGF23 assay was used to measure FGF23 concentration in this study.

Some studies on HHRH cohorts, have shown that heterozygous carriers of the mutation, although asymptomatic, may present with hypercalciuria which puts them at a higher risk of developing nephrocalcinosis [3]. We have shown that all investigated family members had varying degrees of hypercalciuria with *u*Ca:*u*Cr values ranging from 0.15 to 1.05 mol/mol. However, the presence or absence of nephrocalcinosis could not be determined because of the lack of the availability of renal ultrasound. Additionally, an interesting feature of both the clinically affected and unaffected members of the family is that they are consistently shorter and tended to be heavier than their healthy yet undernourished peers. This may well be a function of their familial environment, or perhaps an additional feature of the mutation.

A limitation of this study is that we have only described in silico predictions of the protein containing the novel S168F mutation, located within a highly conserved region, leading to a loss of function of the translated NaPi-IIc protein. Additional mutational analysis is required to determine more detailed effects of this mutation on the protein function and the prevalence of the variant allele needs to be further explored in the general Gambian population. Nevertheless, we have clearly shown that the affected siblings were homozygous in the S168F mutation, whereas the unaffected family members were carriers.

In summary, this study presents a novel mutation in the SLC34AC gene causing HHRH. This is the first kindred of HHRH to be reported in Africa.

## Conflicts of interest

All authors state that they have no conflicts of interest.

## Figures and Tables

**Table 1:** Anthropometry and biochemical local reference data used to create age appropriate Z-scores. Data are presented as mean (SD) or geometric mean (− 1SD, + 1SD) for skewed variables*.

Reference data in age bands (mean SD)					
Variable	2.0–5.9 y (*n* = *10*)	6.0–9.9 y (*n* = *10*)	10.0–13.9 y (*n* = *10*)	14.0–17.9 y (*n* = *10*)	18.0–47.0 y (*n* = *52*)
Ht (cm)	95.3 (10.3)	121.5 (9.8)	146.6 (9.2)	156.6 (9.4)	158.9 (9.7)
Wt (kg)	13.2 (2.6)	20.6 (3.6)	34.5 (7.2)	42.9 (7.9)	58.7 (14.9)
25OHD (nmol/L)	56.3 (18.9)	62.4 (14.7)	55.4 (15.8)	48.3 (13.7)	63.9 (12.0)
TALP (U/L)	299 (65)	325 (67)	298 (86)	298 (135)	125 (58)
1,25(OH)_2_D (pmol/L)	279 (64)	234 (48)	254 (81)	263 (86)	241 (103)
PTH*(pg/mL)	39 (24, 65)	40 (27, 58)	53 (32, 88)	60 (39, 92)	59 (26, 134)
P (mmol/L)	1.59 (0.17)	1.57 (0.19)	1.40 (0.19)	1.40 (0.22)	1.12 (0.18)
Ca (mmol/L)	2.38 (0.08)	2.42 (0.08)	2.36 (0.09)	2.37 (0.09)	2.24 (0.14)
FGF23*(RU/mL)	83 (37, 188)	52 (31, 87)	38 (23, 62)	45 (21, 96)	31 (19, 189)
TmP:GFR (mmol/L)	1.71 (0.27)	1.82 (0.26)	1.64 (0.25)	1.63 (0.29)	1.17 (0.24)
*u*Ca:*u*Cr*(mol/mol)	0.11 (0.03, 0.40)	0.08 (0.02, 0.27)	0.09 (0.04, 0.22)	0.08 (0.02, 0.24)	0.07 (0.02, 0.22)

**Table 2:** Biochemical data of affected (S5*, S2* and S1*) and unaffected (S3 and S4) siblings and their mother. Z-scores were calculated from age-matched data from the local community.

Variable	Affected subjects					Unaffected subjects		
	S5*		S2*		S1 *	S3	S4	Mother
Age (y)	12^a^	18^b^	2^a^	7^b^	4^a^	11	15	35
Ht (cm)	120.4	129.0	86.5	106.1	87.1	128.5	146.8	154.7
*(Z-Score)*	*(− 2.85)*	*(− 2.94)*	*(− 0.85)*	*(− 1.57)*	*(− 0.79)*	*(− 1.97)*	*(– 1.04)*	*(− 0.43)*
Wt (kg)	29.5	46.6	13.7	24.5	13.5	43.9	54.5	62.1
*(Z-Score)*	*(− 0.69)*	*(0.47)*	*(0.19)*	*(1.08)*	*(0.11)*	*(1.30)*	*(1.47)*	*(0.22)*
25OHD (nmol/L)	56.0	58.7	36.6	43.7	64.4	58.3	61.7	91.7
*(Z-Score)*	*(0.02)*	*(0.76)*	*(− 1.04)*	*(− 1.27)*	*(0.43)*	*(0.18)*	*(0.98)*	*(2.32)*
TALP (U/L)	3291	294	689	1269	742	355	145	109
*(Z-Score)*	*(35.00)*	*(− 0.03)*	*(5.95)*	*(14.10)*	*(6.80)*	*(0.66)*	*(− 1.13)*	*(− 0.26)*
1,25(OH)_2_D (pmol/L)	339	341	385	461	–	323	339	486
*(Z-Score)*	*(1.06)*	*(0.91)*	*(1.68)*	*(4.67)*	–	*(0.86)*	*(0.88)*	*(2.37)*
PTH (pg/mL)	14.0	23.0	–	45.1	–	34.0	60.0	29.0
*(Z-Score)*	*(− 2.66)*	*(− 2.29)*	–	*( 0.28)*	–	*(− 0.90)*	*(− 0.01)*	*(− 0.87)*
P (mmol/L)	0.71	0.55	0.67	0.42	0.53	1.30	1.00	1.12
*(Z-Score)*	*(− 3.57)*	*(− 3.89)*	*(− 5.49)*	*(− 5.75)*	*(− 6.33)*	*(− 0.51)*	*(− 1.84)*	*(0.02)*
Ca (mmol/L)	2.46	2.33	2.39	2.40	2.42	2.44	2.39	2.09
*(Z-Score)*	*(1.08)*	*(− 0.22)*	*(0.11)*	*(− 0.26)*	*(0.46)*	*(0.86)*	*(0.46)*	*(− 1.12)*
FGF23 (RU/mL)	57.3	44.6	223.8	64.9	629.9	54.5	32.9	33.5
*(Z-Score)*	*(0.84)*	*(− 0.01)*	*(1.21)*	*(0.43)*	*(2.49)*	*(0.74)*	*(− 0.41)*	*(− 0.52)*
TmP:GFR (mmol/L)	–	0.58	–	0.50	0.63	1.58	1.20	1.27
*(Z-Score)*	–	*(− 3.68)*	–	*(− 5.03)*	*(− 4.00)*	*(− 0.23)*	*(− 1.52)*	*(0.42)*
*u*Ca:*u*Cr (mol/mol)	–	0.20	–	0.38	1.05	0.32	0.15	0.22
*(Z-Score)*	–	*(0.85)*	–	*(1.27)*	*(1.76)*	*(1.40)*	*(0.59)*	*(1.19)*
Thacher X-ray score	4	*F*	8	10	7	–	–	–

^a^ = first presentation, ^b^ = off treatment follow-up, – = not determined, *F* = fused growth plate.

**Table 3:**
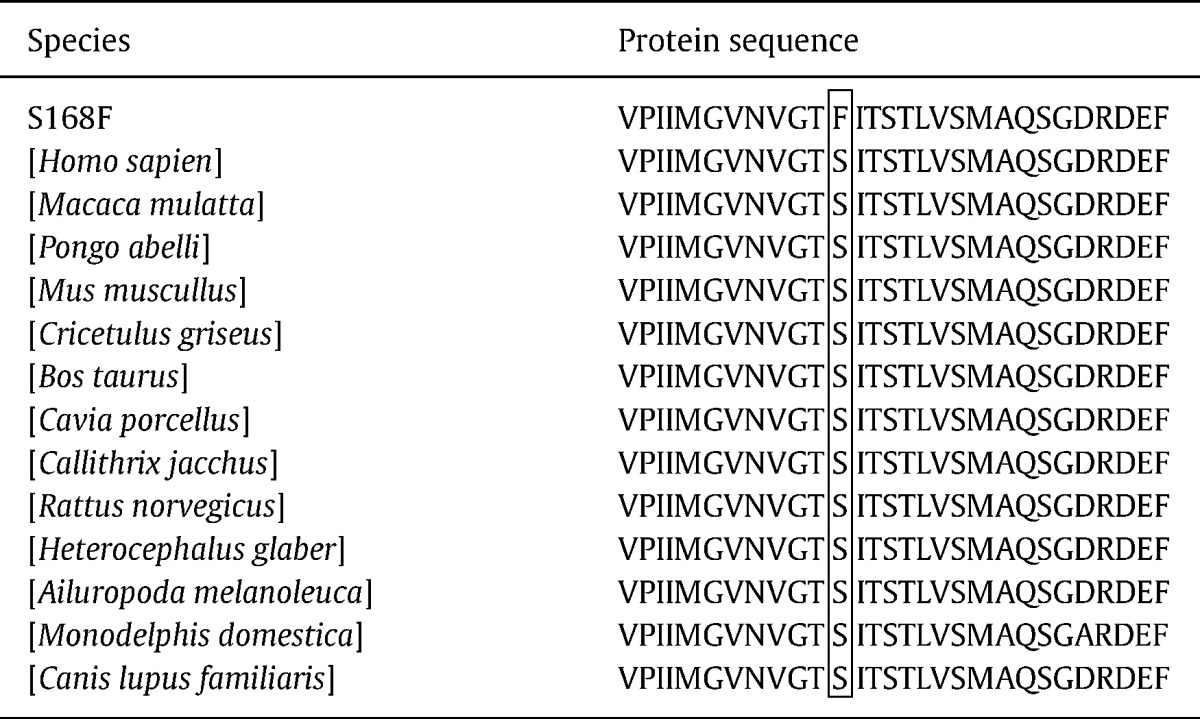
Cross-species protein sequence alignment of flanking regions of the S168F mutation using BLAST®, NCBI database.

## References

[1] Prentice A., Ceesay M., Nigdikar S., Allen S.J., Pettifor J.M. (2008). FGF23 is elevated in Gambian children with rickets. Bone.

[2] Braithwaite V., Jarjou L.M., Goldberg G.R., Jones H., Pettifor J.M., Prentice A. (2011). Follow-up study of Gambian children with rickets-like bone deformities and elevated plasma FGF23: possible aetiological factors. Bone.

[3] Lorenz-Depiereux B., Benet-Pages A., Eckstein G., Tenenbaum-Rakover Y., Wagenstaller J., Tiosano D. (2006). Hereditary hypophosphatemia rickets with hypercalciuria is caused by mutations in the sodium-phosphate cotransporter gene *SLC34A3*. Am J Hum Genet.

[4] Bergwitz C., Roslin N.M., Tieder M., Loredo-Osti J., Bastepe M., Abu-Zahra H. (2006). *SLC34A3* mutations in patients with hereditary hypophosphatemic rickets with hypercalciuria predict a key role for the sodium-phosphate cotransporter NaPi-IIc in maintaining phosphate homeostasis. Am J Hum Genet.

[5] Negri A.L. (2007). Hereditary hypophosphatemias: new genes in the bone-kidney axis. Nephrology.

[6] Thacher T., Fischer P.R., Pettifor J., Lawson J., Manaster B., Reading J.C. (2000). Radiographic scoring method for the assessment of the severity of nutritional rickets. J Trop Pediatr.

[7] Payne R. (1998). Renal tubular reabsorption of phosphate (TmP/GFR): indications and interpretation. Ann Clin Biochem.

[8] Schwarz J.M., Rodelsperger C., Schuelke M., Seelow D., Wagenstaller J., Tiosano D. (2010). MutationTaster evaluates disease-causing potential of sequence alterations. Nat Methods.

[9] Adzhubei I.A., Schmidt S., Peshkin L., Ramensky V.E., Gerasimova A., Bork P., Kondrashov A.S., Sunyaev S.R. (2012). A method and server for predicting damaging missense mutations. Nat Methods.

[10] Farrow E.G., Yu X., Summers L.J., Davis S.I., Fleet J.C., Allen M.R. (2011). Iron deficiency drives an autosomal dominant hypophosphatemic rickets (ADHR) phenotype in fibroblast growth factor-23 (Fgf23) knock-in mice. Proc Natl Acad Sci U S A.

